# Association between RASSF1A Promoter Methylation and Prostate Cancer: A Systematic Review and Meta-Analysis

**DOI:** 10.1371/journal.pone.0075283

**Published:** 2013-09-20

**Authors:** Jincheng Pan, Junxing Chen, Bo Zhang, Xu Chen, Bin Huang, Jintao Zhuang, Chengqiang Mo, Shaopeng Qiu

**Affiliations:** 1 Department of Urology, the First Affiliated Hospital, Sun Yat-sen University, Guangzhou, China; 2 School of Public Health, Sun Yat-sen University, Guangzhou, China; Queen's University Belfast, United Kingdom

## Abstract

Prostate cancer (PCa) remains as one of the most common cause of cancer related death among men in the US. The widely used prostate specific antigen (PSA) screening is limited by low specificity. The diagnostic value of other biomarkers such as RAS association domain family protein 1 A (RASSF1A) promoter methylation in prostate cancer and the relationship between RASSF1A methylation and pathological features or tumor stage remains to be established. Therefore, a meta-analysis of published studies was performed to understand the association between RASSF1A methylation and prostate cancer. In total, 16 studies involving 1431 cases and 565 controls were pooled with a random effect model in this investigation. The odds ratio (OR) of RASSF1A methylation in PCa case, compared to controls, was 14.73 with 95% CI = 7.58–28.61. Stratified analyses consistently showed a similar risk across different sample types and, methylation detection methods. In addition, RASSF1A methylation was associated with high Gleason score OR=2.35, 95% CI: 1.56–3.53. Furthermore, the pooled specificity for all included studies was 0.87 (95% CI: 0.72–0.94), and the pooled sensitivity was 0.76 (95% CI: 0.55–0.89). The specificity in each subgroup stratified by sample type remained above 0.84 and the sensitivity also remained above 0.60. These results suggested that RASSF1A promoter methylation would be a potential biomarker in PCa diagnosis and therapy.

## Introduction

Prostate cancer remains as one of the most common cause of cancer related death among men in the US, with an estimated 29,720 deaths projected in 2013 [1,2]. Prostate cancer is curable if detected early by digital rectal examination and prostate specific antigen (PSA) in the serum [3]. With a sensitivity of 80%, PSA screening significantly increases the early diagnosis of prostate cancer, however the PSA screening has only 20% specificity, which may lead to unnecessary biopsies and overtreatment [4]. Hence diagnosis and management are confounded by the lack of cancer-specific diagnostic techniques to be used during early stages of disease. Novel approaches for the definitive detection and control of this cancer are urgently needed.

Molecular studies have revealed important events in prostate cancer development and progression and the emergence of new biomarkers may improve the diagnosis of prostate cancer [5]. Aberrant DNA methylation of gene promoters is characteristic of cancer cells, and several genes are epigenetically altered in a cancer specific manner. GSTP1 gene promoter methylation is widely characterized by several independent groups and is found to be have diagnostic value in prostate cancer [6,7]. To date, more than one hundred genes are reported as methylation targets in prostate cancer, which may represent a promising method for monitoring the occurrence and progression of cancer [7-9]. Recently, next generation sequencing based studies have also identified additional candidate genes [10]. The chromosomal region 3p21 is subject to frequent loss (heterozygous and homozygous) in multiple tumor types, the RAS association domain family protein 1 A (RASSF1A) gene located in this region, is a putative tumor suppressor which shares high sequence homology with a known mouse protein (Nore1) [11,12]. Studies suggests the role for RASSF1 as the effector that propagates the apoptotic effects of RAS by binding RAS in a guanosine triphosphate dependent manner [13]. Hypermethylation of the CpG islands within the RASSF1A promoter region are the major cause of loss of expression [14]. In many solid tumors, methylation of RASSF1A has been identified and its frequency varies between 30% to 50% [12]. Therefore, RASSF1A methylation affects its tumor suppressor role and is associated with unfavorable prognosis [15,16].

Despite a number of individual studies performed in prostate cancer patients, the diagnostic value of RASSF1A methylation status in prostate cancer and the relationship between RASSF1A methylation and pathological features or tumor stage remains controversial. Therefore, the purpose of this study was to conduct a meta-analysis on the prognostic value of RASSF1A methylation status in prostate cancer, and the relationship between RASSF1A methylation and pathological stage, Gleason score, and PSA level. We also assessed the sensitivity and specificity of RASSF1A methylation in the body fluids and tissues on prostate cancer detection.

## Materials and Methods

### Study Selection

Previously published articles that studied RASSF1 promoter DNA methylation in prostate cancer were identified via an electronic search of PubMed using the following key words; Prostate cancer, PCa, RAS association domain family protein1A, and RASSF1A. Additional studies were found via the reference lists of the identified articles. Only studies published as full-text articles in English were included in this study. The last retrieval was conducted in March 2013.

### Inclusion and Exclusion Criteria

Studies were selected for analysis if they met the following criteria(1): measurement of DNA methylation in one of the following samples: blood, plasma, serum, urine or prostate tissues; (2) the subjects in every study comprised of prostate cancer patients and non-cancer controls; (3) Data was included in the analysis only if the full text of the article was in English. Exclusion criteria were: (1) RASSF1A methylation conducted only in the cell lines; (2) no raw data available or cannot retrieve any raw data; (3) review papers.

### Data Collection

For each study, two independent investigators extracted the following information which include the author’s last name, year of publication, country, race, type of cases and controls. In addition, information was also collected regarding the type of assay method (such as PCR), cancer clinical stage classification, PSA, Gleason score, primers location, CpG location and primers sequence. Details are summarized in Table 1, Table S2 and Table S3. Before we conducted sensitivity and specificity analyses, the case and control methylation data were tabulated. Among the included studies, two categories were assigned as controls: normal controls and patients who had negative biopsies but had other diseases including benign prostatic hyperplasia (BPH). Only biopsy confirmed PCa and high grade prostatic intra-epithelial neoplasia (HGPIN) were treated as cases. Hence the true positive (TP) samples were limited to those that had methylation in the index case, while the false negative (FN) were indicated to have no methylation among the case samples. The same definitions were given for false positive (FP) and true negative (TN) in controls (Table S1).

**Table 1:** Characteristics of studies included in the meta-analysis.

Author	Country	Year	Sample	Methods	Case	Control	Case Met	Case Umet	Control Met	Control Umet
1.Hoque et al	USA	2005	Urine	QMSP	PCa	BPH/OCD or Normal^#^	38 (73.1%)	14	10 (11.0%)	81
2.Roupret et al	France	2007	Urine	QMSP	PCa	Normal^#^	74 (77.9%)	21	3 (7.9%)	35
3.Bastian et al	Portugal	2008	Serum	MSP	PCa	Normal^#^	3 (1.4%)	207	0(0)	35
4.Roupret et al	UK	2008	Blood	QMSP	PCa	BPH	41 (97.6%)	1	5 (22.7%)	17
5.Maruyama et al	USA	2002	Tissue	MSP	PCa	BPH/Normal*(7)	54 (53.5%)	47	5 (15.6%)	27
6.Kang et al	Korea	2004	Tissue	MSP	PCa/HGPIN	Normal^#^	40 (78.4%)	11	0(0)	20
7.Jeronimo et al	Portugal	2004	Tissue	QMSP	PCa/HGPIN	BPH	117 (99.2%)	1	28 (93.3%)	2
8.Yegnasubramanian et al	USA	2004	Tissue	QMSP	PCa	Normal*(12)	70 (95.9%)	3	0(0)	25
9.Singal et al	USA	2004	Tissue	MSP	PCa	BPH	40 (49.4%)	41	8 (19.0%)	34
10.Woodson et al	USA	2004	Tissue	QMSP	PCa/HGPIN	Normal*(11)	23 (67.6%)	11	0(0)	11
11.Florl et al	Germany	2004	Tissue	MSP	PCa	Normal^#^	88 (77.9%)	25	19 (52.8%)	17
12.Bastian et al	Germany	2005	Tissue	QMSP	PCa	BPH	36 (67.9%)	17	4 (28.6%)	10
13.Cho et al	Korea	2007	Tissue	MSP	PCa	BPH	155 (86.6%)	24	7 (23.3%)	23
14.Kawamoto et al	Japan	2007	Tissue	MSP	PCa	BPH	97 (74.0%)	34	12 (18.5%)	53
15.Syeed et al	India	2010	Tissue	MSP	PCa	BPH	17 (34.0%)	33	7 (15.6%)	38
16.Vasiljevic et al	UK/China	2011	Tissue	PYRO	PCa	BPH	44 (91.7%)	4	2 (6.9%)	27

**BPH**, Benign Prostate Hyperplasia; **MSP**, Methlation-Specific PCR; **QMSP**, Quantitative Real time Methylation Specific PCR; **PYRO**, Pyrosequencing; **Met**, methylation; **Umet**,no methylation; **PCa**, Prostate Cancer; **HGPIN**, High Grade Postatic Intraepithelial Neoplasia ;**OCD**, other cancer disease; * Numbers in parenthesis under the control column indicates matched normal tissues; **#** indicates normal prostate tissue from men with no evidence of prostate cancer.

#### Meta-analysis and Statistical Analysis

Odds ratio (OR) with the corresponding 95% CI was used to examine the differences in the frequency of RASSF1A methylation between prostate cancer case and controls. Similarly the association between RASSF1A methylation and prostate cancer pathological stage, Gleason score, and PSA levels were also examined. To assess heterogeneity across the studies, a statistical test for heterogeneity was performed. If the studies were shown to be homogeneous with P>0.05 for the Q-statistics, the summary of OR was calculated by a fixed-effects model, otherwise, a random-effects model was selected. In addition, stratified analyses were also performed by samples and methods, meta-regression was used to study the heterogeneity source, and a sensitivity analysis, by which a single study in the meta-analysis was deleted each time to determine the influence of the individual data set to the overall pooled OR, was performed to assess the stability. The potential publication bias was examined visually by a funnel plot of log [OR] against its standard error (SE), and the degree of asymmetry was tested by Egger’s test. Finally a trim-and-fill method was used to draw stable conclusion. This meta-analysis was performed using the software STATA 11.0. All p-values were based on two-sided tests and p<0.05 was considered statistically significant.

Sensitivity and specificity were analyzed using random-effects models and in this analysis, both the fluid and tissue subgroups were processed. We utilized the summary receiving operating characteristic (S–ROC) curve, to assess whether variation in the threshold definition of a positive result produced an association between sensitivity and specificity values across studies. Estimation of the linear regression of the log–odds ratio from each study on the sum of the logits of the true-positive and false-positive rates enabled S-ROC calculations. When the regression between these quantities was null, an independent analyses of pooled sensitivity and specificity using standard methods for binary data was adopted. Under these circumstances, data were analyzed on the log-odds scale (e.g., for specificities, the effect size used was log(Spec/(1-Spec)). All analyses were conducted in STATA 11.0.

## Results

### Study Characteristics

A total of 16 eligible studies involving 1431 cases and 565 controls were included in the pooled analyses based on our inclusion criteria [15-30]. The characteristics of these studies are summarized in Table 1. Among the 16 studies, 12 studies utilized tissue samples and 4 characterized body fluids such as blood, urine among others. The methylated RASSF1A levels were monitored using either methylation specific PCR (MSP), quantitative methylation specific PCR (QMSP) or pyrosequencing. All cases were from biopsy confirmed PCa or HGPIN, while controls were limited to either BPH, healthy subjects or those who had genitourinary cancer (bladder carcinoma) with a healthy prostate. The samples from 11 studies were mainly from subjects of Caucasian race, while four studies characterized samples from Asian population, one study included subjects from multiple races originating from different continents. Prostate cancers were confirmed pathologically in all the studies.

### Association between RASSF1A promoter methylation and pathological stage, Gleason score, and PSA levels in PCa cases

**Figure 1:**
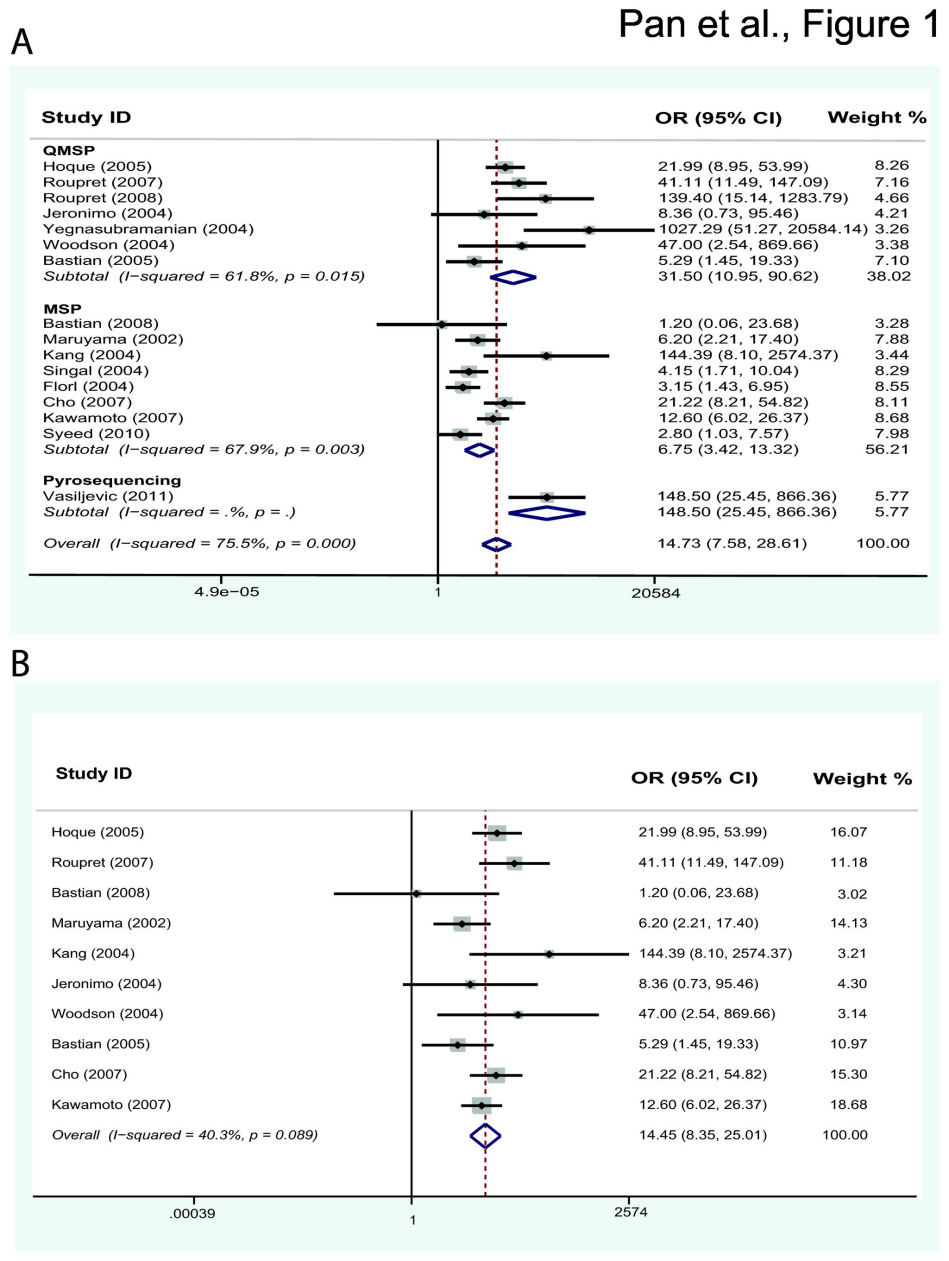
Forest plot showing the association between RASSF1A methylation and prostate cancer. (A) Forest plot of 16 studies showed that RASSF1A methylation was associated with PCa risk as stratified analysis by methods. (B) Forest plot after sensitivity analysis removing 6 studies showed the same risk without heterogeneity (p= 0.089). The diamond symbol represents the overall estimate from the meta-analysis and its CI (confidence interval), while its center is positioned on the value for the overall effect estimate. Diamond’s width depicts the width of the overall CI.

Under the random-effects model, the pooled OR of RASSF1A methylation in prostate cancer cases, compared to non-cancer controls, was 14.73 with 95%CI = 7.58–28.61(Table 2). In the stratified analysis by sample, significantly increased risk was associated with RASSF1A methylation in both fluid samples (OR = 26.27, 95%CI = 7.79–88.61) and in tissues (OR = 12.28, 95%CI = 5.86–25.76). As stratified analysis by method, significantly increased risk was also found in MSP (OR 6.75, 95% CI = 3.42–13.22) and QMSP (OR = 31.50, 95%CI = 10.95–90.62) (Figure 1A). We also conducted an analysis of the relationship between the pathological stage, Gleason score, and PSA levels among PCa cases and RASSF1A methylation. Seven studies [17,19,21-23,25,26] had sufficient information to perform this analysis (Table S2), we found no significant association between groups with the appropriate models except for the Gleason score (OR=2.35, 95% CI: 1.56–3.53, I^2^=32.3%). Details are shown in Table 3.

**Table 2:** Stratification analyses of RASSF1A methylation and prostate cancer risk.

Variables	p^a^	OR (95% CIs)	Heterogeneity Test(I^2^, p-value)
RASSF1A			
Total	16	14.73 (7.58-28.61)	75.5%,0.000
Sample Type			
Fluid	4	26.27 (7.79-88.61)	56.6%,0.075
Tissue	12	12.28 (5.86-25.76)	75.5%,0.000
Method			
QMSP	7	31.50 (10.95-90.62)	61.8%,0.015
MSP	8	6.75 (3.42-13.22)	67.9%,0.003
Pyrosequencing	1	148.50 (25.45-866.36)	NA

**MSP**, Methylation-Specific PCR; **QMSP**, Quantitative real time Methylation Specific PCR; **OR**,Odds Ratio;

**CI**, Confidence Interval; ^a^ Number of studies; **NA**, Not Applicable.

**Table 3:** Association between RASSF1A promoter methylation and Pathological stage, Gleason score, and PSA levels in PCa cases.

Clinicopathology	Category	OR (95% CIs)	Heterogeneity test (I^2^, P-value)	Publication bias test (P-value)	
Gleason score	GS≥7	2.35 (1.56-3.53)	32.3%,0.182	0.40	
	GS<7				
PSA levels	PSA>4	2.19 (0.27-17.76)	67.4%,0.046	0.04	
	PSA≤4				
Pathological stage	Ⅲ&Ⅳ	2.01 (0.77-5.23)	68.4%,0.007	0.73	
	Ⅰ&Ⅱ				

**OR**, Odds Ratio; **CI**, Confidence Interval; **PSA**, Prostate Specific Antigen; **GS** Gleason Score.

The results of meta-regression indicated that the trend in ORs correlated with methylation detection methods, which accounted for part of the heterogeneity (coefficient =−1.69, p=0.024, adjusted R^2^ = 47.41%), However, other factors such as sample types (p=0.525), year of publication (p=0.608), and the origin of the patients (p=0.847) could not explain the heterogeneity. We also performed sensitivity analyses to determine the effects of omitting a single study on the overall effect where six independent studies were found to introduce some source of heterogeneity. Hence when we excluded the six studies that would likely introduce high heterogeneity due to different types of cases and controls, we observed a decrease in heterogeneity (p = 0.089) with stable conclusion (OR = 14.45, 95%CI = 8.35–25.01) (Figure 1B).

We did not observe any publication bias (Egger’s test; p=0.066), and further confirmed this observation by implementing a trim-and-fill method. Meta-analysis with or without the trim-and-fill method did not draw different conclusions, indicating that our results were statistically robust. The Egger’s test suggested the absence of publication bias (P>0. 05) in studies of association between RASSF1A methylation and pathological stage or Gleason score (Table 3).

### Specificity and sensitivity of RASSF1A promoter methylation using different types of samples

**Figure 2:**
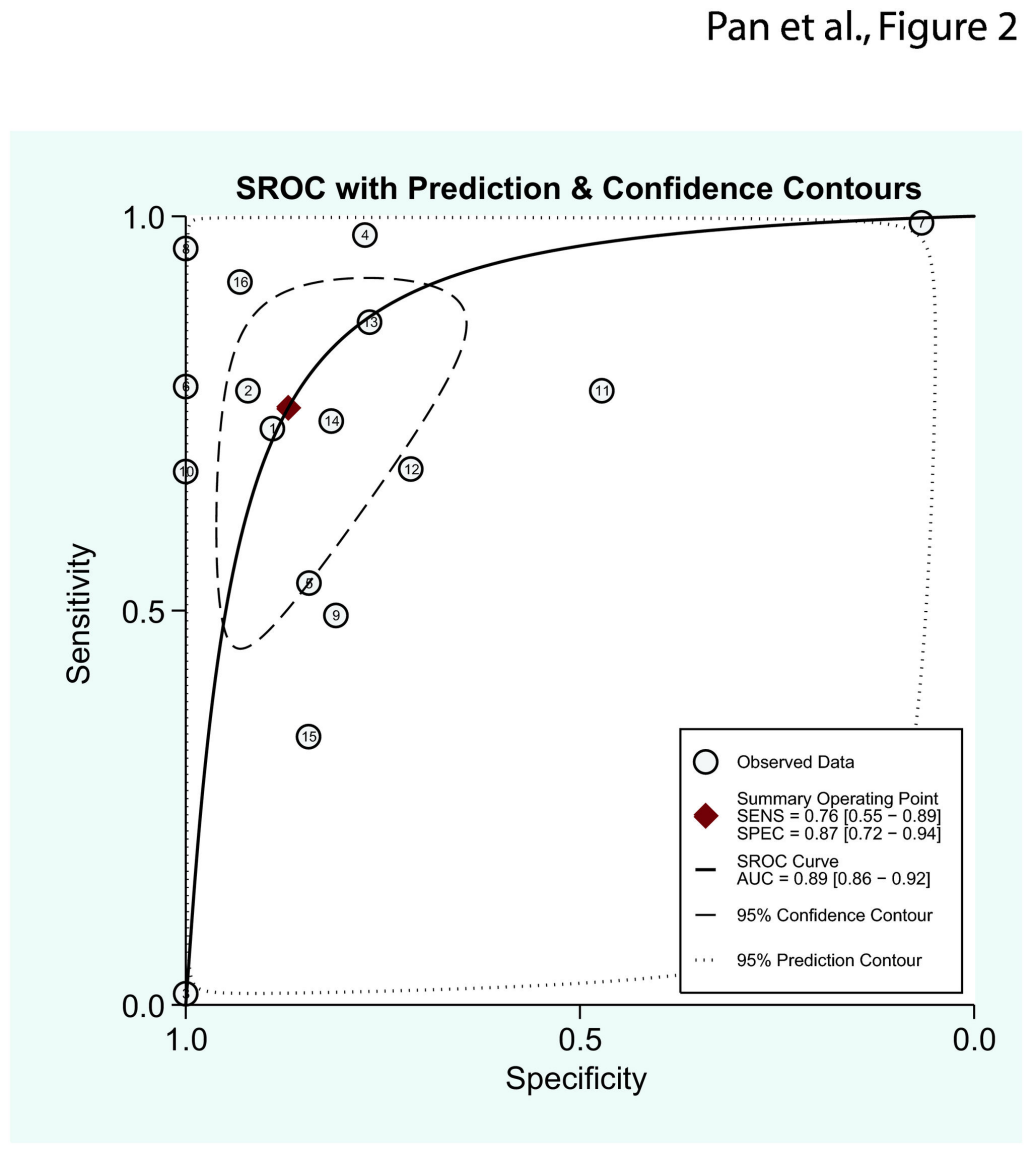
Meta-analysis with the S-ROC curve. SENS: Sensitivity, SPEC: Specificity, AUC: Area under the curve.

The pooled specificity for all included studies was 0.87 (95% CI: 0.72–0.94), and the pooled sensitivity was 0.76 (95% CI: 0.55–0.89). For the traditional biomarker, the sensitivity of PSA varied, but the specificity was generally low at about 20%, which suggested that the RASSF1A methylation test has a much higher specificity than the PSA test. The p-value for heterogeneity was <0.001, indicating a significant heterogeneity. There was no evidence of publication bias (p=0.13). In addition, we classified all samples into two groups according to specimen type (fluid/tissue). Among the fluid studies (urine/blood/plasma), the pooled sensitivity and specificity was 0.60 (95% CI: 0.08–0.96) and 0.93 (95% CI: 0.75–0.98), respectively. For the tissue studies, the pooled sensitivity and specificity was 0.79 (95% CI: 0.64–0.89) and 0.84 (95% CI: 0.63–0.94), respectively and the S–ROC curve is presented in Figure 2.

## Discussion

The results of our meta-analysis showed that RASSF1A methylation in prostate cancer was significantly associated with cancer risk when monitored in urine, blood or tissue samples. It was also associated with increased risk for high Gleason score. The pooled specificity (0.87) of RASSF1A was much higher than the specificity of PSA (20%). In addition the specificity in each subgroup stratified by sample type remained above 0.84 and the sensitivity of RASSF1A also remained high above 0.60. These results suggested that RASSF1A promoter methylation would be a valuable biomarker in diagnosing PCa.

DNA methylation is a common mechanism for inactivating tumor-suppressor genes in cancers [10]. Monitoring aberrant methylation patterns have aided in the detection of tumor cells in clinical specimens such as tissue biopsies or body fluids. RASSF1A is a putative tumor suppressor gene and plays an important role in the regulation of different types of human tumors. Previous reports demonstrated that genetic variation of RASSF1A affect prostate cancer susceptibility and the frequency of RASSF1A methylation was found to be significantly higher in patients’ group compared with control [13]. To further confirm RASSF1A promoter methylation status in PCa patient’s diagnosis, we carried out a meta-analysis of 16 studies involving 1431 cases and 565 controls to derive a more precise estimation of the association. Our results suggested that RASSF1A methylation is a potential risk factor for PCa as detected both in body fluid and tissues. The frequency of RASSF1A methylation in PCa cases was 14.73 times higher than that in control individuals. In addition, due to differences between cases and controls between studies, we performed sensitivity analyses and removed select studies to make the data more homogeneous and observed no major changes to our conclusion. Importantly, frequent methylation of RASSF1A gene showed significant associations with Gleason score, although it did not correlate with pathological stage or PSA levels in this study.

As shown previously, the PSA test had varying sensitivity and poor specificity (about 20%) [1]. Tests that provide higher specificity rather than sensitivity and complement PSA testing is the current requirement. Promisingly, our results show that, RASSF1A methylation testing has a potential to complement PSA screening due to its high specificity. Here we believe a sequential testing model will be more helpful where PSA test will be initially used to identify patients with elevated PSA levels, followed by the RASSF1A methylation test to increase true positivity. Patients with negative RASSF1A test can be placed on watchful waiting, while individuals with positive results can be recommended for biopsies. Thus sequential testing will greatly reduce the unnecessary biopsy recommendations based solely on PSA testing.

The present study has several limitations. First, the heterogeneity in the methods employed such as MSP, Q-MSP and pyrosequencing to monitor gene promoter methylation. Based on studies conducted, it will be helpful if a consensus could be arrived for a standard testing. Sequencing based techniques are generally considered to have superior performance as compared to methodologies that employ PCR alone. The rapidly gaining momentum in using next generation sequencing (NGS) assays to monitor genomic and epigenomic changes will certainly usher in advancements in this area [31]. One can foresee development of comprehensive diagnostic tests that will simultaneously measure transcript expression and DNA methylation changes of a panel of biomarkers in addition to identifying genetic aberrations such as gene fusions and copy number variations for each patient [32]. The current study along with others helps in shortlisting biomarkers of great potential that will be given special attention while analyzing NGS results which usually produces a long list of methylated regions. Next, the diverse sample types employed in these studies which include plasma, serum, urine, whole blood and tissues is a potential source of heterogeneity. Here again development of high performance robust assays with high sensitivity and specificity may help overcome this in the near future.

## Conclusion

This meta-analysis has demonstrated that RASSF1A methylation in prostate cancer was associated with cancer risk across different study populations. RASSF1A is a promising biomarker for screening and identifying PCa especially when combined with the PSA test to bring down the rate of unnecessary biopsies. Future studies with larger sample cohort and novel assays based on next generation sequencing approaches will help in further strengthening our observations.

## Supporting Information

Table S1 **Characteristics of studies included in the meta-analysis.**
(XLSX)

Table S2 **Details of studies included in the meta-analysis: Association between RASSF1A promoter methylation and pathological stage, Gleason score, and PSA levels.**
(XLSX)

Table S3 **Summary of primer sequences used in methylation study of RASSF1A.**
(XLSX)
